# Predicting Life Expectancy Using Veterans Affairs Electronic Health Record Data

**DOI:** 10.1093/geroni/igaa057.561

**Published:** 2020-12-16

**Authors:** Alexandra Lee, Bocheng Jing, Sun Young Jeon, John Boscardin, Sei Lee

**Affiliations:** 1 University of California, San Francisco, San Francisco, California, United States; 2 San Francisco VA Medical Center, San Francisco, California, United States

## Abstract

Electronic health records (EHRs) are a rich source of health data that could be used to create individualized life expectancy predictions to aid in clinical decision-making for long-term preventative treatments, such as cancer screening. Few previous studies have incorporated all possible predictors from the EHR. We aimed to screen and incorporate a large number of possible predictors from EHR data into a life expectancy (LE) prediction equation. Using the national Veteran’s Affairs (VA) EHR databases, we identified all patients aged 50+ with a primary care visit during 2005 and assessed demographics, diseases, medications, laboratory results, healthcare utilization, and vital signs during the one year prior to the visit. Mortality follow-up was complete through 2017. We used an 80% random sample for model development and a 20% random sample for model validation. We used a Gompertz survival model with backwards selection to identify approximately 100 variables for the final LE prediction equation. In 1,263,595 VA patients, the mean age was 68 years and the majority were male (94%) and white (87%). During 12 years of follow-up, 602,576 (47.7%) died. Of 930 predictors from the EHR, 99 were included in the LE prediction equation. Harrell’s C-statistic was 0.7705 (95%CI: 0.7693, 0.7718). The model estimated 10-year life expectancy with sensitivity of 81.6% (81.4%, 81.8%) and specificity of 68.8% (68.5%, 69.1%). In conclusion, we developed an LE prediction equation from hundreds of predictors in the VA EHR with good discrimination and calibration that may help clinicians weigh the potential benefit of long-term preventative treatments.

